# From Amyloid to Synaptic Dysfunction: Biomarker-Driven Insights into Alzheimer’s Disease

**DOI:** 10.3390/cimb47080580

**Published:** 2025-07-22

**Authors:** Luisa Agnello, Caterina Maria Gambino, Anna Maria Ciaccio, Francesco Cacciabaudo, Davide Massa, Anna Masucci, Martina Tamburello, Roberta Vassallo, Mauro Midiri, Concetta Scazzone, Marcello Ciaccio

**Affiliations:** 1 Institute of Clinical Biochemistry, Clinical Molecular Medicine, and Clinical Laboratory Medicine, Department of Biomedicine, Neurosciences, and Advanced Diagnostics, University of Palermo, 90127 Palermo, Italy; luisa.agnello@unipa.it (L.A.); caterinamaria.gambinoi@unipa.it (C.M.G.); francesco.cacciabaudo@unipa.it (F.C.); davide.massa@unipa.it (D.M.); anna.masucci@unipa.it (A.M.); martina.tamburello@unipa.it (M.T.); roberta.vassallo@unipa.it (R.V.); concetta.scazzone@unipa.it (C.S.); 2 Department of Laboratory Medicine, University Hospital “Paolo Giaccone”, 90127 Palermo, Italy; 3 Department of Health Promotion, Mother and Childcare, Internal Medicine and Medical Specialties (PROMISE), University of Palermo, 90127 Palermo, Italy; annamaria.ciaccio@unipa.it; 4 Forensic Medicine Unit, Department of Health Promotion, Mother and Childcare, Internal Medicine and Medical Specialties (PROMISE), University of Palermo, 90127 Palermo, Italy; mauro.midiri@unipa.it

**Keywords:** diagnostic criteria, Alzheimer’s disease, biomarkers, plasma, CSF, blood, ATX(N) framework

## Abstract

Alzheimer’s disease (AD) is the most prevalent neurodegenerative disorder and represents a major public health challenge. With increasing life expectancy, the incidence of AD has also increased, highlighting the need for early diagnosis and improved monitoring. Traditionally, diagnosis has relied on clinical symptoms and neuroimaging; however, the introduction of biomarkers has revolutionized disease assessment. Traditional biomarkers, including the Aβ42/Aβ40 ratio, phosphorylated tau (p-Tau181, p-Tau217, and p-Tau231), total tau (t-tau), and neurofilament light chain (NfL), are fundamental for staging AD progression. Updated guidelines introduced the ATX(N) model, which extends biomarker classification to include additional promising biomarkers, such as SNAP-25, YKL-40, GAP-43, VILIP-1, progranulin (PGRN), TREM2, IGF-1, hFABP, MCP-1, TDP-43, and BDNF. Recent advancements have allowed for the detection of these biomarkers not only in CSF but also in plasma and neuron-derived exosomes, offering less invasive and more accessible diagnostic options. This review explores established and emerging biomarkers and emphasizes their roles in early diagnosis, patient stratification, and precision medicine.

## 1. Introduction

The increase in life expectancy, along with medical and public health advancements, has significantly reshaped the epidemiological landscape of prevalent diseases, which is shifting from infectious diseases to neurodegenerative diseases (NDs), with Alzheimer’s disease (AD) being the most common [1]. AD, characterized by a progressive decline in memory, language, and autonomy, represents a major challenge for healthcare systems, necessitating targeted long-term care strategies. Currently, one in nine individuals over the age of 65 has AD, and its prevalence is projected to increase significantly in the coming decades [2]. Given its increasing prevalence, AD has been recognized as a global health priority, leading to an exponential growth in research over the past decades. AD follows a progressive trajectory, beginning with subtle, undetectable brain changes and advancing to cognitive impairment and physical decline [3]. Notably, the preclinical phase, identifiable through neuroimaging and biomarkers, can last for decades before clinical symptoms appear. The AD continuum is classified into stages by frameworks such as the National Institute on Aging (NIA) and the Alzheimer’s Association (AA) research criteria, which delineate the preclinical, mild cognitive impairment (MCI), and dementia phases. Key genetic factors, such as the APOE ε4 allele, and modifiable risk factors, including cardiovascular health and lifestyle, significantly impact the speed of disease progression. The pathogenesis of AD begins 20 years or more before symptoms appear, suggesting a significant window for intervention in disease progression [2]. In this scenario, biomarkers play a critical role in allowing for the early detection of the mechanisms underpinning AD and, therefore, the early diagnosis of AD. Addressing modifiable risk factors that could slow or delay cognitive decline, such as hypertension, physical activity, and cognitive stimulation, has shown potential to delay the onset of cognitive impairment [4]. Additionally, the detection of individuals in the early stages of AD allows for the identification of patients eligible for clinical trials and thus promotes research on treatment.

A thorough understanding of AD pathophysiology is essential for the rational selection of biomarkers.

Extracellular amyloid plaques, composed primarily of aggregated Aβ peptides and intracellular neurofibrillary tangles consisting of phosphorylated tau (pTau), are the pathological hallmarks of AD. Neuroinflammation mediated by microglia and astrocytes, synaptic dysfunction, and tau hyperphosphorylation contribute to AD progression [5].

**Figure 1:**
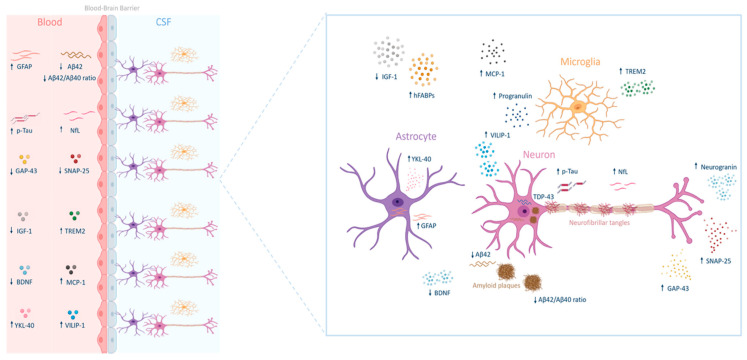
Biomarkers of Alzheimer’s disease across the blood–brain Barrier. Glial fibrillary acidic protein (GFAP), phosphorylated tau protein (p-TAU), growth-associated protein 43 (GAP-43), insulin-like growth factor 1 (IGF-1), brain-derived neurotrophic factor (BDNF), chitinase-3-like protein 1 (YKL-40), amyloid β 42 peptide (Aβ42), amyloid β 40 peptide (Aβ40), neurofilament light chain (NfL), synaptosomal-associated protein 25 (SNAP-25), triggering receptor expressed on myeloid cells 2 (TREM2), monocyte chemoattractant protein-1 (MCP-1), visinin-like protein 1 (VILIP-1), heart-type fatty acid-binding proteins (hFABPs), and TAR DNA-binding protein 43 (TDP-43).

This review aims to explore the molecular biomarkers of AD (Figure 1), briefly describing well-established core biomarkers, and focusing on the most recent discoveries. Finally, because biomarkers have revolutionized the diagnostic criteria for AD, we provide an overview of their evolution.

## 2. Core Biomarkers

### 2.1. β-Amyloid Peptide

Aβ is a small protein (4 kDa) that results from the cleavage of amyloid precursor protein (APP), a transmembrane molecule produced by neurons, vascular cells, and blood cells, including platelets and astrocytes [6]. APP undergoes sequential proteolysis by β-secretase and γ-secretase to generate Aβ peptides that exist as multiple isoforms, primarily Aβ40 and Aβ42. Aβ40 and Aβ42 differ in two amino acid residues and their metabolism, physiological functions, toxicities, and aggregation mechanisms. Aβ40 is the most abundant isoform in the brain. However, Aβ42 tends to aggregate more easily and contributes to the formation of amyloid plaques, which are associated with neurodegeneration in AD. The increased aggregation propensity of Aβ42 is attributed to its structural properties, which enhance fibril formation and promote plaque accumulation. Aβ42 aggregation progresses through several stages, starting from small, soluble units such as dimers and trimers and eventually forming larger, insoluble fibrils and plaques that impair neuronal function [7].

Oligomeric Aβ species are neurotoxic, disrupt synaptic transmission and trigger inflammatory responses. Amyloid plaques are a hallmark of AD, and their presence in the brain is associated with neuronal damage and cognitive decline [8]. Plaques contribute to synaptic dysfunction, neuroinflammation, and oxidative stress, exacerbating neurodegeneration. According to the new 2024 diagnostic criteria for AD, isoforms Aβ40 and Aβ42 are crucial for identifying and classifying the disease [9]. The differential roles of Aβ40 and Aβ42 in AD pathophysiology have led to their incorporation into biomarker panels for early detection. Although Aβ42 is involved in amyloid plaque formation, Aβ40 does not play a direct role in AD pathogenesis [5]. However, the Aβ42/Aβ40 ratio is useful for normalizing individual variations in the Aβ levels. Thus, the Aβ42/Aβ40 ratio is a critical diagnostic tool for adjusting for individual baseline differences in Aβ production and clearance. Typically, patients with AD have reduced Aβ42 and Aβ42/Aβ40 ratio, reflecting impaired clearance and increased amyloid deposition [10].

### 2.2. Tau Protein

Tau protein is essential for stabilizing microtubules in the neurons of the central nervous system (CNS) [11]. Under pathological conditions, such as AD, Tau undergoes hyperphosphorylation, causing its detachment from microtubules and the formation of intracellular toxic aggregates known as neurofibrillary tangles (NFTs). These tangles, along with Aβ plaques, constitute the main neuropathological hallmarks of AD [12]. Hyperphosphorylated Tau disrupts neuronal function by interfering with microtubule dynamics, contributing to cell cycle dysregulation, synaptic dysfunction, and neuronal death [13].

This pathological process is further exacerbated by a reduction in the activity of Protein Phosphatase 2A (PP2A), a major tau phosphatase frequently impaired in AD. Tau biomarkers evolve dynamically throughout the disease course and, according to updated criteria, are stratified into two main categories, T1 and T2, based on their temporal changes and clinical relevance [14]. T1 biomarkers include phosphorylated tau species, such as p-Tau181, p-Tau217, and p-Tau231, which are pathologically altered during the early stages of the disease, concurrent with amyloid PET abnormalities. T2 biomarkers, in contrast, comprise imaging markers, such as tau PET, and specific tau-derived fragments measurable in cerebrospinal fluid (CSF) and plasma, including Microtubule-Binding Repeat Tau243 (MTBR-Tau243). These biomarkers emerge at more advanced stages of the disease and are strongly correlated with the onset of clinical symptoms in AD. Thus, while T1 biomarkers are indicative of an early pathological response, T2 biomarkers reflect the late-stage tau aggregation characteristic of AD, which is often detected in conjunction with tau PET imaging. To improve diagnostic accuracy, p-Tau biomarkers in the CSF were also evaluated as ratios, such as p-Tau181/Aβ42 and t-Tau/Aβ42 [15]. Some plasma-based p-tau assays have demonstrated strong diagnostic performance as independent biomarkers [16]. It has been proposed that the first detectable pathological tau variant in the CSF and plasma of patients with AD is p-Tau181, followed sequentially by p-Tau217, p-Tau231, pT205, MTBR-Tau243, and eventually non-phosphorylated tau fragments [14]. Core biomarkers follow a structured trajectory that mirrors the pathological evolution of AD. Amyloid biomarkers (e.g., Aβ42/Aβ40 ratio and amyloid PET) manifest early and are instrumental in identifying the preclinical phase of AD. In contrast, tau biomarkers (e.g., p-Tau, t-Tau, and MTBR-Tau243) become relevant at later disease stages, reflecting tau pathology and progressive neuronal degeneration. These biomarkers offer a comprehensive assessment of AD pathology, aiding in disease staging and differential diagnosis.

## 3. Prognostic Biomarkers

### 3.1. Neurofilaments

Neurofilaments (Nfs) are essential structural proteins in neurons, particularly within axons, that contribute to cytoskeletal integrity and axonal transport [17]. They comprise three subunits: neurofilament light (NfL), medium (NfM), and heavy (NfH). In the CNS, Nfs interact with α-internexin, whereas in the peripheral nervous system (PNS), they associate with peripherin. Among them, NfL is the most abundant. Upon neuronal degeneration, they are released into the extracellular space and, consequently, their levels increase in the CSF and blood. NfL has been recognized as a nonspecific biomarker of neurodegeneration in several neurological diseases [18,19]. Although NfL effectively differentiates AD from healthy aging, it is less specific in distinguishing among neurodegenerative disorders [20]. In AD, blood NfL levels increase with disease progression, correlating with cognitive decline and brain atrophy [21,22]. Therefore, it serves as a prognostic biomarker for AD.

### 3.2. Glial Fibrillary Acidic Protein

Glial Fibrillary Acidic Protein (GFAP) is a key structural component of the astrocyte cytoskeleton and plays a critical role in glial cell activation and response to neuroinflammation [23]. It is involved in multiple cellular processes, including cell motility and migration, the remodeling of glial structures during mitosis with tightly regulated RNA and protein expression, exocytosis, vesicle trafficking, synapse formation, neuronal plasticity, neurite growth, neuronal germination, maintenance of CNS myelination, and blood–brain barrier (BBB) integrity [24].

Recent studies have supported the use of GFAP as a biomarker for AD. It can reliably detect AD at an early stage and differentiate it from other dementias [25]. It is closely linked to the fundamental pathological mechanisms of AD, including amyloid and tau deposition, hypometabolism, and brain atrophy, thus underlining the importance of early astrocyte activation in disease progression. A study conducted by Pereira et al. emphasized that plasma GFAP serves as an early and specific biomarker for AD, correlating strongly with Aβ deposition and cognitive decline [26]. Furthermore, GFAP levels are significantly elevated in individuals with familial AD. O’Connor et al. reported that plasma GFAP levels were considerably higher in mutation carriers, both before and after symptom onset, than in non-carriers. Interestingly, GFAP levels begin to change up to 16 years before clinical manifestation, making GFAP a promising early biomarker. In symptomatic mutation carriers, GFAP levels were more than three times higher than in non-carriers. In contrast, the levels of presymptomatic individuals approximately double, indicating a progressive increase as the disease advances [27].

In 2024, GFAP was incorporated into the updated diagnostic and staging criteria for AD, reinforcing its relevance in clinical practice for managing patients with AD [9].

Interestingly, GFAP in plasma has demonstrated superior sensitivity and specificity compared to that in CSF. A cross-sectional study conducted in 2021 reported that plasma GFAP levels were elevated in both preclinical and symptomatic AD stages, with values surpassing those observed in the CSF [28]. Moreover, plasma GFAP exhibited greater accuracy than CSF GFAP in differentiating Aβ-positive individuals from Aβ-negative individuals, even during the preclinical phase. A 2023 study further examined GFAP levels in the serum and CSF of patients with AD, dementia with Lewy bodies (DLB), and healthy controls, revealing sex-related differences in CSF GFAP levels exclusively in patients with AD. However, these differences were observed in the serum across all groups. Additionally, as age increases, the differences in GFAP levels between clinical groups become less distinct, suggesting that aging may influence biomarker interpretation in neurodegenerative disease monitoring [29].

Collectively, these findings emphasize the growing role of GFAP as a highly relevant biomarker for AD.

### 3.3. Neurogranin

Neurogranin (Ng) is a synaptic protein involved in the regulation of synaptic function and plasticity [30]. In AD, Ng levels in the CSF are elevated and correlate with disease progression, whereas plasma Ng levels do not show a strong correlation, limiting their reliability in clinical assessments. Several studies have reported significantly higher Ng levels in the CSF of patients with AD than in those with other neurodegenerative diseases [31,32]. Ng is closely linked to Aβ plaque formation and cognitive decline, highlighting its role as a specific biomarker for AD. Additionally, elevated CSF Ng levels correlate with neurodegenerative markers, indicating a strong association with synaptic loss [33,34,35,36]. Ng levels in the CSF are also associated with brain atrophy and amyloid accumulation, making them useful for early-stage AD detection [37].

Furthermore, a study by Mattsson et al. demonstrated that combining t-Tau, Ng, and NfL improved AD diagnostic accuracy compared with individual biomarkers [38]. A 2020 meta-analysis confirmed that CSF Ng levels are significantly higher in AD patients than in healthy controls and MCI patients, but no significant difference was observed between AD and dementia with Lewy Bodies (DLB), indicating limitations in disease specificity [39]. Moreover, MCI patients who progressed to AD had higher Ng levels in the CSF than those with stable MCI, suggesting that Ng can help differentiate progressive MCI from stable cases but may not distinguish AD from other NDs, such as frontotemporal dementia (FTD) or DLB. A recent study analyzed Ng levels in post-mortem brain tissue from AD patients, older adults, and middle-aged individuals [40]. The results demonstrated that increased Ng levels in the CSF reflect a corresponding loss of Ng in the brains of patients with AD, further supporting its use as a biomarker for AD and possibly for cognitive decline associated with normal aging.

In conclusion, the evidence strongly supports CSF neurogranin (Ng) as a valuable biomarker for AD, given its association with synaptic loss, amyloid accumulation, and disease progression. Elevated Ng levels in the CSF can help to differentiate AD from healthy aging and monitor disease progression, particularly when identifying progressive MCI cases. However, its specificity remains limited, as it does not clearly distinguish AD from other neurodegenerative disorders, such as DLB or FTD.

## 4. Evolution of Diagnostic Criteria for Alzheimer’s Disease

**Figure 2:**
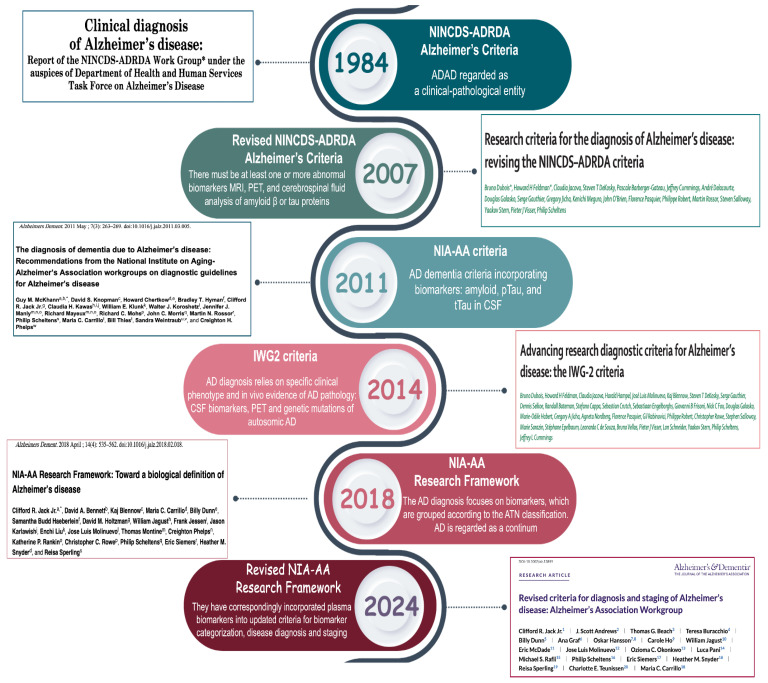
Timeline of Alzheimer’s disease diagnostic criteria evolution (1984–2024). This timeline summarizes the key milestones in the development of the diagnostic criteria for AD, from the 1984 NINCDS-ADRDA criteria to the most recent 2024 revision [14,41,42,43,44,45]. Over the years, the definition of AD has evolved from a purely clinical construct to a biologically driven model. The 2011 and 2014 updates introduced the use of CSF biomarkers and imaging techniques to detect amyloid and tau pathologies. The 2018 NIA-AA Research Framework formalized AT(N) classification, categorizing biomarkers into three main groups: amyloid (A), tau (T), and neurodegeneration (N). In 2024, the revised NIA-AA criteria incorporated blood-based biomarkers and introduced an expanded ATX(N) model. The “X” category includes emerging biomarkers that capture additional pathological processes, such as neuroinflammation, synaptic dysfunction, and blood–brain barrier damage.

The diagnostic criteria for AD have evolved over the years, moving from an approach based solely on clinical symptoms to a more complex model that integrates biomarkers, advanced imaging techniques, and a deeper understanding of the underlying pathology (Figure 2). Recent diagnostic frameworks emphasize the role of fluid biomarkers, genetic risk profiling, and neuroimaging in enhancing the diagnostic accuracy and allowing for earlier detection.

In 1984, the National Institute of Neurological Disorders and Stroke (NINDS) and Alzheimer’s Disease and Related Disorders Association (ADRDA) introduced the first diagnostic criteria [41]. These criteria marked a significant step forward by standardizing AD diagnosis and distinguishing between probable, possible, and definitive AD based on clinical presentation and neuropathological confirmation.

AD is defined as progressive dementia, usually observed in older age but sometimes also in middle-aged individuals. The diagnosis was based on clinical observations and the exclusion of other causes of dementia. Neuropathological examination remains the gold standard for confirming AD, with hallmark lesions including amyloid plaques and neurofibrillary tangles. Probable AD is characterized by a progressive decline in memory and cognition, whereas possible AD encompasses cases with atypical symptomatology or comorbidities that could contribute to cognitive impairment.

The emergence of biomarker-based frameworks has refined AD diagnosis, allowing for earlier detection and more precise classification of the disease stages.

In 2007, an international group of experts (IWG) reviewed these criteria and introduced an approach that included the use of biomarkers and advanced imaging technologies to identify diseases at earlier stages [42]. This revision seeks to diagnose AD not only in the presence of clear symptoms but also during its early stages. This emphasizes the importance of episodic memory as the main symptom, along with abnormal biomarkers detected through Magnetic Resonance Imaging (MRI), Positron Emission Tomography (PET) imaging, and CSF analysis.

The integration of MRI, PET, and CSF biomarkers into diagnostic workflows has improved early detection, particularly in individuals with MCI that may progress to AD.

The concept of a disease continuum has also been recognized, introducing MCI as an intermediate stage between normal aging and dementia. MCI is described as a mild cognitive decline that does not significantly affect daily activities but may represent a prodromal condition for AD, especially in its amnestic form. This classification reinforced the idea that AD is not an abrupt-onset disease but rather a continuum, with a gradual transition from preclinical stages to full-blown dementia.

In 2011, the NIA-AA published new criteria that combined clinical criteria and biomarkers [43]. This update integrated both clinical symptomatology and biomarker evidence, enabling a more precise stratification of AD stages. This revision outlines the diagnosis of AD in three main stages: preclinical, characterized by the presence of abnormal biomarkers but no obvious clinical symptoms; MCI due to AD, which includes mild cognitive decline with pathological biomarkers; and full-blown dementia, defined as cognitive decline that interferes with daily activities. Although biomarkers are considered essential for improving diagnostic accuracy, they are mainly recommended for research and not for routine clinical practice.

In 2014, the criteria were further updated by the IWG with the publication of the IWG-2 guidelines [44]. This revision reinforced the concept of AD as a clinical–biological disease, emphasizing the central role of biomarkers in all disease stages.

The disease is described as a continuum with three main stages: the preclinical stage, in which biomarkers help identify at-risk individuals without obvious symptoms; the prodromal stage, corresponding to MCI with specific memory changes; and the full dementia stage, characterized by severe cognitive decline. This classification aligns with previous models but places greater emphasis on biomarker-driven diagnosis to improve early detection and intervention.

The main biomarkers include Aβ deposits detected through PET or reduced Aβ42 in the CSF, and tau protein abnormalities observed in the CSF or with PET. The importance of signs of neurodegeneration, such as brain atrophy visible on MRI or reduced glucose metabolism detected by Fluorodeoxyglucose Positron Emission Tomography (FDG-PET), has also been recognized. This biomarker-based approach allows for earlier and more accurate diagnoses, although accessibility to advanced technologies remains challenging in many settings.

In 2018, the NIA-AA Research Framework introduced a new biomarker-based system to classify the disease spectrum and improve participant selection in clinical trials [45]. This framework formalized the ATN classification, offering a standardized method for categorizing AD based on biological markers rather than clinical symptoms alone.

The ATN system classifies biomarkers into three categories, as indicated by the letter A, which refers to biomarkers of Aβ deposition, such as Aβ42 and Aβ42/Aβ40 ratio; T, which refers to biomarkers of pathological tau, such as p-tau; and N, which refers to neurodegeneration, such as total tau (t-tau). This classification helps differentiate AD from other NDs and defines eight distinct biological profiles along the disease spectrum depending on the combination of biomarkers. While the ATN system is helpful for outlining AD, other factors, such as inflammation or mixed pathologies, may contribute to cognitive decline. Despite its utility, the ATN system does not capture all pathological processes involved in AD, necessitating further refinement to incorporate additional factors, such as neuroinflammation and vascular contributions. Finally, by 2024, the criteria for diagnosing AD will be further refined [14].

The latest revision aimed to improve disease staging by integrating a wider range of biological markers and refining their clinical applications. This classification acknowledges the dynamic nature of biomarker progression, distinguishing markers indicative of early pathology from those associated with advanced neurodegeneration. Core biomarker 1, such as amyloid PET scans or the Aβ42/40 ratio in the CSF, helps identify the disease in its earliest phases, even when no symptoms are present. Core biomarker 2, such as tau PET scans, is essential for monitoring the progression and severity of disease in later stages. This distinction facilitates personalized diagnostic and therapeutic strategies, allowing for tailored interventions based on the disease stage. Another major innovation is the new biological staging system, which describes the progression of the disease in four phases: Phase A involves the initial biological changes; Phase B reflects early visible alterations; Phase C includes intermediate changes; Phase D represents advanced modifications of the disease. This model offers a more granular perspective of disease progression, enabling the more precise stratification of patients based on their biomarker profiles.

**Figure 3:**
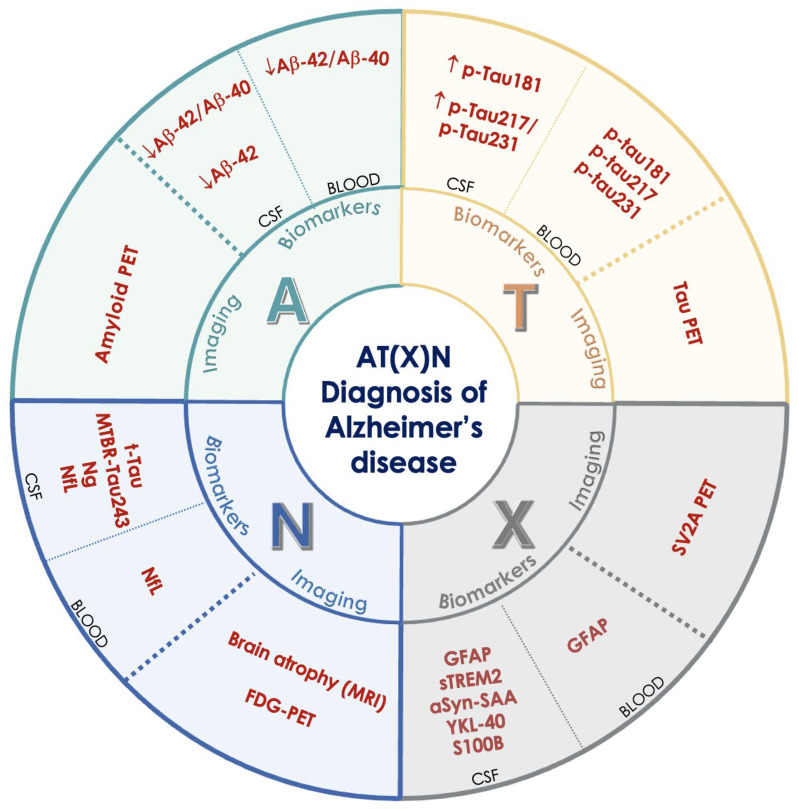
The AT(X)N framework for the biological diagnosis and staging of Alzheimer’s disease. The framework includes three main categories of biomarkers: A (amyloid) for β-amyloid deposition (Aβ42, Aβ42/Aβ40 ratio, amyloid PET), T (tau) for tau pathology (p-Tau181, p-Tau217, p-Tau231, tau PET), and N (neurodegeneration) for neuronal damage (t-Tau, MTBR-Tau243, NfL, brain atrophy on MRI, FDG-PET). The X category includes emerging biomarkers that reflect other disease mechanisms, such as neuroinflammation (GFAP, sTREM2, YKL-40), synaptic dysfunction, and blood–brain barrier damage (e.g., sPDGFRβ), which are not captured by the traditional ATN model. β-amyloid 40 (Aβ40), β-amyloid 42 (Aβ42), cerebrospinal fluid (CSF), glial fibrillary acidic protein (GFAP), phosphorylated tau (isoforms 181, 217, 231) (p-TAU), total tau (t-Tau), alpha-synuclein seeding amplification assay (alfaSyn-SAA), FDG positron emission tomography (FDG-PET), neurogranin (Ng), neurofilament light chain (NfL), synaptosomal-associated protein 25 (SNAP-25), soluble triggering receptor expressed on myeloid cells 2 (sTREM2), microtubule-binding region tau 243 (MTBR-Tau243), YKL-40, S100 calcium-binding protein B (S100B), synaptic vesicle glycoprotein 2A positron emission tomography (SV2A PET).

In addition to core biomarkers, non-core biomarkers have been introduced. The ATX(N) classification system is an evolution of the AT(N) framework, which introduces an additional category, “X,” to incorporate emerging biomarkers that capture other relevant pathophysiological processes in AD (Figure 3). Specifically, this category includes the following: i) neuroinflammation, detected by biomarkers such as GFAP, which reflects astrocytic activation; ii) synaptic dysfunction, detected by biomarkers such as neurogranin, which is indicative of synaptic integrity; and iii) BBB integrity, assessed by soluble PDGFRβ.

These biomarkers can be measured in the CSF, in the blood, or through advanced imaging, and are grouped based on the type of sample or diagnostic method used.

The integration of these additional biomarkers aims to provide a more comprehensive understanding of the multifaceted pathology of AD, enhance the diagnostic accuracy, and facilitate the development of targeted therapeutic strategies. This approach emphasizes the biological definition of AD, moving beyond clinical symptomatology.

## 5. Promising Biomarkers in Alzheimer’s Disease

**Figure 4:**
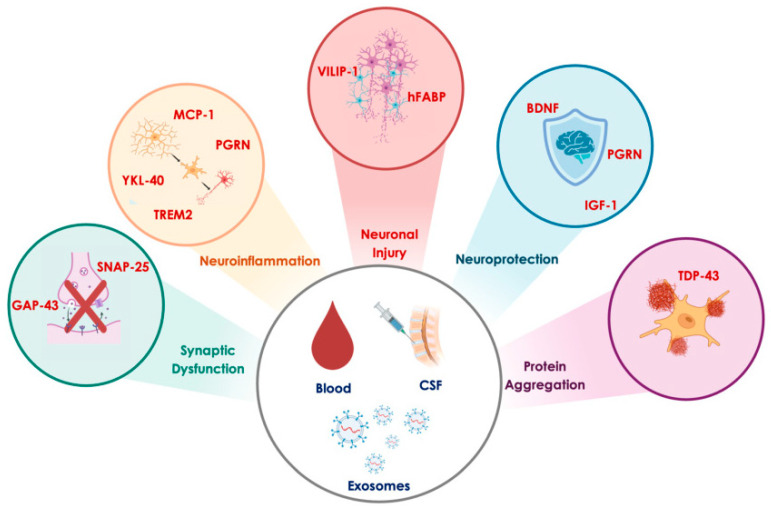
Promising biomarkers reflecting key pathophysiological processes in Alzheimer’s disease. The biomarkers of synaptic dysfunction include growth-associated protein 43 (GAP-43) and synaptosomal-associated protein 25 (SNAP-25). Biomarkers of neuroinflammation include monocyte chemoattractant protein-1 (MCP-1), progranulin (PGRN), triggering receptor expressed on myeloid cells 2 (TREM2), and chitinase-3-like protein 1 (YKL-40). Biomarkers of neuronal injury include visinin-like protein 1 (VILIP-1) and heart-type fatty acid binding protein (hFABP). Biomarkers of neuroprotection comprise brain-derived neurotrophic factor (BDNF), progranulin (PGRN), and insulin-like growth factor 1 (IGF-1). Finally, the biomarker of protein aggregation is TAR DNA-binding protein 43 (TDP-43).

In recent years, significant progress has been made in the understanding of the molecular mechanisms underlying AD. However, the early diagnosis and effective monitoring of this disease remain challenging. In this context, identifying new biomarkers could enhance disease management and support rapid diagnosis and targeted treatment. Newly emerging biomarkers reflect the various pathological processes involved in AD, including synaptic dysfunction, neuroinflammation, neuronal damage, and neuroprotection. Among the most promising candidates are synaptosomal-associated protein 25 (SNAP-25), chitinase 3-like protein 1 (YKL-40), growth-associated protein 43 (GAP-43), visinin-like protein 1 (VILIP-1), progranulin (PGRN), triggering receptor expressed on myeloid cells 2 (TREM2), insulin-like growth factor 1 (IGF-1), fatty acid-binding proteins (FABPs), monocyte chemoattractant protein-1 (MCP-1), TAR DNA-binding protein 43 (TDP-43), and brain-derived neurotrophic factor (BDNF) [46,47] (Figure 4). Many of these biomarkers can be detected in CSF, blood, or neuron-derived exosomes, offering less invasive and more accessible alternatives to traditional diagnostic tools such as neuroimaging and lumbar puncture. Notably, some of these biomarkers show alterations in the early and preclinical stages of the disease and hold promise for earlier diagnosis. This section explores each of these biomarkers and discusses their characteristics, their roles in the disease, and their clinical potential (Table 1).

**Table 1:** Promising biomarkers in Alzheimer’s diseases.

Biomarkers	Biological Matrix	Clinical Interpretation in AD	References
**SNAP-25**	CSF, neuron-derived exosomes	Biomarker of early synaptic dysfunction. It increases in CSF during preclinical AD and its levels correlate with tau and APOE ε4. It can predict AD 5–14 years before symptoms.	[48,49,50,51,52,53,54,55,56,57,58,59,60,61]
**YKL-40**	CSF, blood	Biomarker of neuroinflammation. It increases in early AD and MCI, and its levels correlate with tau and Aβ.	[62,63,64,65,66,67,68,69,70,71,72]
**GAP-43**	CSF, neuron-derived exosomes	Biomarker of synaptic plasticity and degeneration. Its levels increase in MCI-to-AD conversion and correlate with t-Tau and p-Tau.	[46,49,54,73,74,75,76,77,78,79,80,81,82,83,84,85]
**VILIP-1**	CSF, blood	Biomarker of neuronal injury and calcium-mediated neurodegeneration. Its levels correlate with brain atrophy and cognitive decline.	[39,86,87,88,89,90,91,92,93,94]
**PGRN**	CSF	Biomarker of microglial activation and neuroinflammation. It modulates Aβ and tau pathology. The diagnostic utility is debated but it is implicated in disease progression.	[95,96,97,98,99,100,101,102,103,104,105,106,107,108]
**TREM2**	CSF, blood	Biomarker of microglial activation. It has an early increase followed by a decline.	[109,110,111,112,113,114,115,116,117,118,119,120,121,122,123,124,125,126,127,128]
**IGF-1**	CSF, blood	Neurotrophic and neuroprotective factor. Its altered signaling is linked to cerebral insulin resistance and Aβ/tau pathology.	[129,130,131,132,133]
**hFABP**	CSF	Early biomarker of neurodegeneration, involved in lipid metabolism and neuronal injury. It correlates with inflammation and atrophy.	[134,135,136,137,138,139,140,141,142,143,144,145,146]
**MCP-1**	CSF, blood	Chemokine involved in neuroinflammation and BBB permeability. It is associated with Aβ deposition and tau pathology.	[147,148,149,150,151,152,153,154]
**TDP-43**	Post-mortem brain (no validated fluid biomarker yet)	Pathological protein aggregates in >50% of AD brains. It is associated with cognitive decline, tau interaction, and *APOE4* genotype.	[155,156,157,158,159,160,161,162,163,164,165,166,167,168,169,170,171]
**BDNF**	CSF, blood	Neurotrophin involved in synaptic plasticity, neuroprotection, and APP metabolism. Its levels are reduced in AD and linked to cognitive decline.	[172,173,174,175,176,177,178,179,180,181,182,183,184,185,186,187,188]

SNAP-25, synaptosomal-associated protein 25; YKL-40, chitinase-3-like protein 1; GAP43, growth-associated protein 43; VILIP1, visinin-like protein 1; PGRN, progranulin; TREM2, triggering receptor expressed on myeloid cells 2; IGF-1, insulin-like growth factor 1; hFABP, heart-type fatty acid-binding protein; MCP1, monocyte chemoattractant protein 1; TDP43, transactive response DNA-binding protein 43; BDNF, brain-derived neurotrophic factor; AD, Alzheimer’s disease; CSF, cerebrospinal fluid.

### 5.1. Synaptosomal-Associated Protein 25

SNAP-25 is a presynaptic protein involved in the release of neurotransmitters during synaptic transmission [48] (Table 2). It forms a coiled-coil complex called soluble NSF attachment protein receptor (SNARE) with syntaxin-1 and synaptobrevin, facilitating synaptic vesicles to fuse with the presynaptic membrane in response to calcium signals [49]. Beyond its presynaptic role, SNAP-25 regulates postsynaptic receptor trafficking, including N-methyl-D-aspartate (NMDA) and kainate receptors, influencing dendritic spine density and morphology [48]. Alterations in SNAP-25 levels impair synaptic transmission, leading to cognitive and behavioral deficits. Moreover, SNAP-25 plays a role in neurite outgrowth and long-term potentiation (LTP), essential processes for synaptic plasticity and cognitive functions [50]. The first significant evidence linking SNAP-25 to AD emerged in 2014 [51]. The authors demonstrated that SNAP-25 levels in CSF are significantly elevated in AD patients compared to healthy controls, even in the early stages of the disease. This suggested that SNAP-25 could serve as a valuable biomarker for the early diagnosis of AD. Subsequent studies confirmed these findings, reporting similar increases in CSF SNAP-25 levels during the preclinical stages of AD [52]. Interestingly, elevated SNAP-25 levels are also observed in Parkinson’s disease (PD) and Creutzfeldt–Jakob disease (CJD), highlighting its potential relevance across a variety of neurodegenerative conditions. In 2019, Agliardi et al. explored SNAP-25 levels in neuron-derived exosomes (NDEs) from blood [53]. They found that SNAP-25 levels are lower in the blood NDEs of AD patients compared to healthy controls, reflecting synaptic changes in the brain. This contrasted with findings in CSF, where SNAP-25 levels are elevated, suggesting that SNAP-25 might be released through different mechanisms in blood versus CSF. Lower SNAP-25 in blood exosomes correlated with cognitive decline, providing an accessible biomarker for monitoring AD progression. Subsequent studies have confirmed this difference: SNAP-25 is lower in blood NDEs but higher in the CSF of AD patients [54]. Additionally, when combined with other biomarkers like GAP-43 and Ng, SNAP-25 showed potential in predicting AD up to 5–7 years before symptom onset, making it a promising early diagnostic tool. A meta-analysis conducted by Roveta systematically evaluated SNAP-25 as a potential fluid biomarker for AD [55]. Focusing on CSF measurements, the study reported significantly higher SNAP-25 concentrations in AD patients. This reinforced the hypothesis that SNAP-25 elevation correlates with synaptic damage and cognitive decline. However, the meta-analysis revealed moderate to high heterogeneity across studies, suggesting variability in patient populations, disease stages, or assay methodologies. Liu et al. conducted a similar meta-analysis and identified a strong correlation between SNAP-25 and tau biomarkers (t-Tau and p-Tau), further supporting the idea that SNAP-25 reflects synaptic damage [56]. These findings encouraged the integration of SNAP-25 into the AT(N) classification framework for AD biomarkers, specifically under the neurodegeneration (“N”) category. The study also revealed that APOE ɛ4 carriers exhibited higher SNAP-25 levels in CSF, indicating potential genetic interactions influencing synaptic damage. A later study confirmed this result [57]. Recent evidence has shown that SNAP-25 can be quantified using the Simoa method and mass spectrometry (IP-MS) [58]. Both methods are highly compatible and provide very similar results, confirming their equivalence in terms of accuracy and reliability. This suggests that both methods can be successfully used in clinical settings to distinguish between patients with Aβ+ pathology and those without (Aβ−). However, while the Simoa test for CSF analysis was successful, plasma measurements remained difficult due to low SNAP-25 concentrations. The same group of researchers later observed that the increase in SNAP-25 continues throughout the entire AD continuum, from the preclinical stage (cognitively intact individuals with Aβ+) to dementia [59]. This suggests that SNAP-25 reflects synaptic changes caused by Aβ pathology before tau accumulation and neurodegeneration occur. Another study showed that SNAP-25 changes in CSF are detectable 12–14 years before clinical symptoms emerge [60]. This indicates that synaptic dysfunction precedes noticeable cognitive decline, positioning SNAP-25 as a marker of early pathological processes, including microglial activation and synaptic damage. Experimental models have further highlighted the importance of SNAP-25 [61]. The authors demonstrated that SNAP-25 decreased in the hippocampus of rats treated with streptozotocin (STZ), a substance that mimics AD-like pathology. Rats receiving STZ directly into the brain (ICV) showed SNAP-25 reduction within one week, while intraperitoneal (IP) STZ administration led to decreases after three weeks. SNAP-25 loss worsened over time, correlating with increased oxidative stress, cognitive deficits, and impaired learning. This finding suggests that SNAP-25 depletion might contribute to synaptic damage and memory loss in AD. In summary, these studies highlight SNAP-25 as a useful and early biomarker for AD. The presence of SNAP-25 in CSF, NDEs, and experimental models underscores its role in synaptic dysfunction and neurodegeneration. SNAP-25 not only reflects synaptic damage but also offers diagnostic and prognostic value across the AD continuum. The relationship between SNAP-25, tau protein, and APOE ɛ4 enhances its importance in the diagnosis and monitoring of AD.

### 5.2. Chitinase-3-like Protein 1 

YKL-40, also known as chitinase-3-like protein 1 (CHI3L1), is a 40 kDa glycoprotein that binds heparin and chitin [62] (Table 3). It is primarily secreted by astrocytes and plays a key role in the inflammatory responses within the CNS. YKL-40 has been identified as a promising biomarker for AD owing to its strong association with disease progression and neurodegeneration.

Studies have shown that CSF YKL-40 levels are elevated in patients with AD compared with cognitively healthy controls, correlating with disease severity [63,64]. It is particularly linked to cognitive decline, especially in the early stages of the disease [65]. Moreover, YKL-40 levels in the CSF correlate with other key AD biomarkers, such as tau and Aβ [66,67]. This suggested that YKL-40 precedes Aβ deposition and plays a role in the early stages of AD pathogenesis. A study by Hampel et al. highlighted the value of YKL-40 as part of a biomarker panel (including Aβ1-42, t-tau, p-tau, NfL, and Ng) to enhance diagnosis and monitor disease progression [68].

In studies involving YKL-40 inhibitors, it was found that inhibiting this protein reduced neuroinflammation, Aβ accumulation, and memory deficits in animal models, ultimately improving cognitive function [69,70]. Interestingly, the deletion of YKL-40 in animal models led to a reduction in amyloid burden but an increase in inflammation, indicating that YKL-40 may have a dual role in neurodegeneration and inflammation.

Longitudinal studies have demonstrated that YKL-40 levels in the CSF increase over time in individuals with MCI and AD but remain stable in healthy controls [71]. This highlights its potential as a predictive marker of disease progression. Additionally, YKL-40 has been evaluated in other matrices, such as plasma, where it is elevated compared to controls, but its utility remains limited compared to that of other AD biomarkers [72].

Overall, YKL-40 is a promising biomarker for AD, particularly for monitoring early stages of the disease and tracking its progression. Although further validation is required to establish its role as a standalone diagnostic marker, combining YKL-40 with other biomarkers could significantly improve early diagnosis and monitoring of AD.

### 5.3. Neuromodulin or GAP-43

GAP-43, also known as neuromodulin, is a presynaptic protein that plays a key role in synaptic plasticity and neuronal development (Table 4). It is primarily found in the hippocampus, entorhinal cortex, and neocortex, where it helps regulate axon growth, synaptic formation, and learning-related processes [73,74]. When phosphorylated by protein kinase C (PKC), GAP-43 interacts with synaptic proteins such as SNAP-25 and synaptophysin, facilitating synaptic vesicle recycling [49]. GAP-43 is increasingly being recognized as a biomarker for AD diagnosis and progression given its strong link to synaptic degeneration. Studies have demonstrated that CSF GAP-43 levels are significantly elevated in patients with AD compared to those in healthy controls and other neurodegenerative diseases [46,49]. The increase in GAP-43 is particularly evident in patients with high t-Tau and p-Tau levels, while its negative correlation with Aβ42 suggests a connection between synaptopathy, amyloid accumulation, and tau pathology [75,76]. Furthermore, GAP-43 levels in patients with positive AD biomarkers are comparable to those in patients with confirmed neuropathological diagnoses, reinforcing its diagnostic relevance [75].

GAP-43 has also been identified as a predictor of disease progression in MCI patients. Longitudinal studies have indicated that higher CSF GAP-43 levels predict conversion from MCI to AD, and are associated with accelerated cognitive decline [54,77,78]. Additionally, exosomal GAP-43, which is strongly correlated with CSF levels, may serve as an accessible blood-based biomarker, offering a new avenue for early diagnosis [54]. Combining GAP-43 with other synaptic biomarkers, such as Ng and SNAP-25, enhances the diagnostic accuracy, allowing for the better differentiation of AD from other dementias [79,80].

The role of GAP-43 in synaptic plasticity and neurodegeneration has been further validated by post-mortem studies, showing reduced expression in the frontal cortex and hippocampus of patients with AD, with a negative correlation with senile plaque density [46]. However, certain hippocampal subregions show increased GAP-43 expression, suggesting a potential compensatory mechanism in early AD [46,81]. The connection between GAP-43 and tau pathology was further investigated in a recent study, showing that higher CSF GAP-43 levels are linked to faster tau propagation, likely facilitated by transsynaptic transmission [82]. This suggests that amyloid-induced synaptic hyperactivity may promote tau release at synapses, contributing to neurofibrillary tangle formation. Genetic factors also influence GAP-43 expression, with APOE ε4 carriers exhibiting higher CSF GAP-43 levels and a more rapid increase over time, indicating greater early synaptic vulnerability and cognitive decline acceleration [76,83]. Imaging studies support this link, demonstrating that increased GAP-43 is associated with hippocampal and medial temporal lobe atrophy, as well as neuronal metabolism changes detected via FDG-PET [80,83]. Recently, GAP-43 was proposed for inclusion in the AT(N) framework for AD staging as higher protein levels were found in A + T + N+ subjects, suggesting an early transition to dementia [84]. Moreover, GAP-43 levels have been correlated with brain microstructural changes observed via diffusion tensor imaging (DTI), suggesting that this protein may indicate early structural degeneration. Interestingly, animal model studies have revealed that GAP-43 reduction extends beyond the brain, affecting other organs, such as the heart, suggesting a potential link between synaptic dysfunction and extraneural AD symptoms [85]. Despite its high diagnostic and prognostic potential, some open questions remain, including the need for comparisons with other synaptic markers and a deeper understanding of its role in disease progression [80,82]. The increasing focus on GAP-43 underscores its value in improving the early diagnosis of AD and in enhancing our understanding of neurodegeneration.

### 5.4. Visinin-like Protein 1

Visinin-like protein 1 (VILIP-1 or VLP-1) is a calcium-sensing protein belonging to the neuronal calcium sensor (NCS) family [39] (Table 5). It plays crucial roles in synaptic transmission and plasticity. Changes in its expression and CSF release have been linked to several NDs, including AD. Recent studies have suggested that VILIP-1 could serve as a promising biomarker for AD diagnosis and prognosis, as its levels are significantly elevated in patients with AD compared to healthy controls, correlating with cognitive decline and brain atrophy [86]. One of the first studies to identify VILIP-1 as a potential biomarker of neuronal damage was conducted by Lee et al., who reported significantly higher CSF VILIP-1 levels in patients than in controls [87]. These findings were later confirmed, showing that VILIP-1 levels correlate with the rate of cognitive decline in patients with AD, and that the VILIP-1/Aβ42 ratio has a diagnostic value comparable to that of t-Tau and p-Tau181 [88]. This study suggests that VILIP-1 can predict cognitive decline even in cognitively normal individuals, indicating its potential role in the preclinical phase of AD. A meta-analysis confirmed that VILIP-1 is significantly increased in the CSF of AD patients compared to controls [39]. However, the authors highlighted substantial variability across studies, likely due to differences in the detection methods and participant selection. Additional research by Mroczko et al. suggested that VILIP-1 levels may have a prognostic value in AD progression [89]. Specifically, they found that patients with MCI who later developed AD exhibited higher CSF VILIP-1 levels than those with stable MCI, supporting the hypothesis that VILIP-1 can help identify individuals at risk of AD conversion. Similarly, neuroimaging studies have revealed correlations between VILIP-1 levels and hippocampal and entorhinal cortex atrophy, two brain regions that are particularly vulnerable to AD [90]. These findings suggest that VILIP-1 expression reflects neuronal loss in these areas. More recently, longitudinal studies have described a distinct trend in VILIP-1 levels throughout the disease course [91]. The authors reported that although VILIP-1 levels were initially high in patients with AD, they tended to decline as the disease progressed. A similar pattern was observed for other neuronal injury biomarkers such as SNAP-25 and Ng. This suggests that VILIP-1 release into the CSF is the highest during the early stages of neurodegeneration and declines as fewer viable neurons remain. While most studies have analyzed VILIP-1 in the CSF, some researchers have explored its presence in plasma as a potential noninvasive biomarker [92]. They observed higher plasma VILIP-1 levels in patients with AD than in controls, although the difference was less pronounced than that in the CSF. This suggests that while plasma VILIP-1 may have diagnostic potential, further validation is needed to confirm its clinical utility.

Another key aspect is the association between VILIP-1 and APOE ε4 genotype, which is the strongest genetic risk factor for AD [93]. One study found that APOE ε4 carriers exhibit higher VILIP-1 CSF levels than non-carriers, suggesting a potential interaction between amyloid metabolism and calcium-mediated neurodegeneration. Despite strong evidence supporting VILIP-1 as an AD biomarker, some limitations remain. First, its specificity to AD is still debated, as elevated VILIP-1 levels have also been observed in other NDs, such as DLB and CJD [94]. Additionally, variability in measurement methods and diagnostic criteria across studies may affect reliability. Further research on larger cohorts using standardized methodologies is necessary to confirm its clinical applicability and to define its diagnostic and prognostic role in AD.

### 5.5. Progranulin

PGRN is a glycoprotein involved in cell growth regulation, immune responses, and lysosomal functions [95] (Table 6). It is mainly expressed in neurons and microglia, and plays a key role in neuronal survival and inflammation control. Loss-of-function mutations in GRN, which reduce PGRN levels, are a major genetic cause of FTD [96]. Various studies suggest that PGRN may also be linked to AD [97,98].

Increased PGRN levels have been observed in microglia surrounding amyloid plaques in patients with AD, suggesting their involvement in neuroinflammation and neurodegeneration [99]. The authors demonstrated that PGRN levels are reduced in the early stages of AD, before amyloid deposition, and that PGRN deficiency exacerbates cognitive impairment, synaptic dysfunction, and neuronal loss. Moreover, PGRN overexpression reduces plaque load, improves neuronal survival, and mitigates cognitive deficits.

Similarly, mouse models of AD have shown that deletion of the GRN gene increases Aβ deposition, intracellular tau accumulation, and microglial activation, whereas PGRN overexpression exerts neuroprotective effects, reduces plaque formation, and improves memory [100]. Longitudinal studies have shown that CSF-PGRN levels increase as AD progresses, correlating with neurodegenerative markers such as t-Tau, p-Tau181P, and cognitive decline, although their role as biomarkers remains debated [101,102].

Tauopathy models have demonstrated that PGRN deficiency accelerates tau phosphorylation and intraneuronal accumulation, suggesting a direct role of PGRN in modulating tau pathology [103]. However, studies in humans have yielded mixed results; while CSF-PGRN levels are higher in AD patients than in healthy controls, several cross-sectional studies have found no clear diagnostic value [102,104,105,106]. Suárez-Calvet et al. proposed that CSF-PGRN should be considered a marker of microglial function rather than a specific AD biomarker, as it correlates with other microglial markers such as sTREM2 [102].

A longitudinal study found that in patients with suspected non-AD pathology (SNAP)who exhibited tau pathology without Aβ deposition, higher CSF-PGRN levels were linked to faster Aβ plaque formation, suggesting that PGRN plays a role in neurodegeneration beyond AD [107]. Another study demonstrated that PGRN reduction affects lysosomal metabolism by regulating glucocerebrosidase (GCase), leading to glucosylceramide (GlcCer) accumulation, which in turn promotes tau and α-synuclein aggregation, indicating that PGRN may be involved in both AD and synucleinopathies [108].

While PGRN’s role as an AD biomarker remains uncertain, its increased CSF levels during disease progression, correlation with cognitive decline and neurodegeneration markers, and involvement in protein aggregation and neuroinflammation suggest that it could be a key player in AD by modulating microglial response and Aβ/tau clearance.

### 5.6. Triggering Receptor Expressed on Myeloid Cells 2

TREM2 is a membrane-bound protein that is predominantly expressed in microglia and other myeloid cells [109] (Table 7). It is crucial to phagocytosis, the modulation of inflammatory responses, and the regulation of brain lipid metabolism [110,111]. TREM2 significantly affects the progression of neurological disorders, particularly AD [112,113,114]. In 2013, two independent studies identified rare TREM2 variants, including the R47H mutation, as major risk factors for late-onset AD (LOAD). These variants were found to increase AD risk to a degree comparable to the APOE ε4 allele [115,116]. Since then, there has been extensive research focused on elucidating how TREM2 modulates amyloid and tau pathology, and its potential role as a biomarker for AD diagnosis and progression monitoring [112,117,118]. TREM2 is a membrane-bound protein that is predominantly expressed in microglia and other myeloid cells [119]. It is crucial to phagocytosis, the modulation of inflammatory responses, and the regulation of brain lipid metabolism [110,111]. TREM2 significantly affects the progression of neurological disorders, particularly AD [112,113,114].

TREM2 plays a key role in microglial responses to Aβ accumulation [119,120]. Its deficiency or mutations, such as R47H, impair microglial clustering around amyloid plaques, leading to diffuse and less compact deposits associated with increased neuritic damage [112]. Additionally, pathogenic variants such as R47H and R62H reduce TREM2’s binding affinity to Aβ, weakening its interaction with plaques and compromising amyloid degradation [121]. TREM2 also plays a crucial role in tau pathology. Studies on CSF have shown that soluble TREM2 (sTREM2), generated by proteolytic cleavage of the membrane-bound form, increases in the early AD stages and correlates with p-tau levels [122,123]. Mouse models with TREM2 deletion exhibit an accelerated spread of pathological tau, suggesting a potential regulatory role of TREM2 in tauopathy progression [117].

The relationship between TREM2 and APOE, another major genetic risk factor for AD, has provided new insights into AD pathogenesis [124,125]. APOE serves as a ligand for TREM2 and enhances microglial phagocytosis and Aβ clearance. TREM2 mutations weaken this interaction, leading to uncontrolled amyloid accumulation and altered microglial activity. Mouse models have shown that TREM2 deletion reduces cortical APOE expression, exacerbating neurodegeneration [117].

The growing interest in TREM2 as an AD biomarker stems from the fact that sTREM2 can be measured in CSF and the blood. CSF sTREM2 levels increase in the early stages of AD, particularly following Aβ deposition, indicating an ongoing microglial response to neurodegeneration. However, at advanced stages of AD, sTREM2 levels decline, reflecting progressive microglial dysfunction [121,123]. A recent meta-analysis reported that plasma sTREM2 levels were significantly higher in patients with AD than in healthy controls, supporting its potential as a non-invasive diagnostic biomarker [112].

The modulation of TREM2 is being explored as a therapeutic approach for AD. TREM2-activating antibodies are under investigation for boosting microglial activation and improving Aβ phagocytosis [126,127]. Conversely, at later AD stages, inhibiting TREM2 might help mitigate neuron damage linked to tau pathology, as evidenced in mouse models treated with antisense oligonucleotides [117,128].

Although TREM2 holds promise as a biomarker for AD, further studies are necessary to establish the predictive value of sTREM2 and clarify its role in different disease stages.

### 5.7. Insulin-like Growth Factor-1

IGF-1 is a peptide hormone belonging to the insulin family of proteins and is essential for maintaining homeostasis by regulating cell growth, synaptic plasticity, and glucose metabolism [129] (Table 8). Additionally, IGF-1 plays a crucial role in the CNS by supporting neuronal survival and modulating neuroinflammation [130]. Recent studies have suggested that altered IGF-1 signaling contributes to AD pathogenesis, making it a potential biomarker for disease diagnosis and monitoring [129,131,132].

There is growing evidence that AD may be considered a form of ‘type 3 diabetes’ (T3D), owing to its association with cerebral insulin resistance and IGF-1 dysfunction [129]. Reduced circulating IGF-1 levels correlate with increased amyloid burden in mouse models of AD and cognitive decline. Alterations in the insulin/IGF-1 pathway, including reduced receptor expression and abnormal phosphorylation of proteins in the PI3K/Akt and RAS/RAF/MAPK pathways, have been identified in the brains of AD patients, suggesting a strong connection between metabolic dysfunction and neurodegeneration [133]. In AD mouse models, RNA sequencing (RNA-seq) analysis has shown that mice raised in germ-free (GF) conditions exhibit less impaired IGF-1 signaling than those raised in specific pathogen-free (SPF) environments, implying a potential role of the gut microbiota in modulating brain insulin signaling and neuroinflammation. Additionally, C/EBPβ, a transcription factor activated in AD microglia, disrupts IGF-1 signaling and increases inflammation through the production of pro-inflammatory molecules derived from arachidonic acid, which may accelerate neurodegeneration [131].

Another key aspect is IGF-1’s role in BBB regulation and neuroinflammation. Changes in IGF-1 production can compromise BBB integrity, allowing pro-inflammatory molecules to infiltrate the brain and exacerbating neurodegeneration [132]. Furthermore, lower IGF-1 expression in the brain has been associated with increased Aβ deposition and higher tau phosphorylation, which are hallmark processes of AD pathology [131].

Owing to its strong association with neuroinflammation, amyloid metabolism, and synaptic dysfunction, IGF-1 represents a potential AD biomarker. However, further studies are required to validate their usefulness in AD patients.

### 5.8. Heart-Type Fatty Acid-Binding Protein

FABPs are a family of proteins involved in the binding, transport, and metabolism of long-chain fatty acids [134] (Table 9). Multiple FABP isoforms are expressed in the human brain, including heart-type FABP (hFABP, FABP3), brain-type FABP (B-FABP, FABP7), and epidermal FABP (E-FABP, FABP5) [135]. These proteins play crucial roles in membrane fluidity, intracellular signaling, and neuronal protection [136]. While their importance in brain development is well established, their involvement in NDs such as AD has only recently been explored.

Among the FABP family members, hFABP (FABP3) has been the most extensively studied for its role in neurodegeneration. While its precise origin in the CSF remains unclear, hFABP is highly expressed in the brain, with levels second only to those in the muscle tissue (Protein Atlas). Post-mortem analyses have revealed significant reductions in hFABP levels in the frontal, temporal, occipital, and parietal cortices of patients with AD and Down syndrome (DS), suggesting a link between altered lipid metabolism, membrane instability, and neuronal dysfunction [137].

The first evidence of hFABP as a potential biomarker for AD came from a study that showed its elevated levels in the CSF, supporting its diagnostic value [138]. Subsequent research confirmed that hFABP is one of the most altered analytes in the CSF of patients with AD, reinforcing its role in neurodegeneration [139]. Notably, these studies suggested that hFABP alterations precede Aβ42 changes, implicating lipid metabolism and vascular dysfunction in early AD pathogenesis.

Further studies have examined the association between hFABP and CSF Aβ42 levels, although no direct correlation with cognitive decline has been reported [140]. A meta-analysis by Olsson et al. found a moderate association between CSF hFABP levels and AD, suggesting a potential diagnostic role, although less pronounced than that of t-Tau [141].

Longitudinal studies have shown that elevated hFABP levels in the CSF predict conversion from MCI to AD, supporting their utility in tracking disease progression [142]. Moreover, brain imaging studies have demonstrated that CSF hFABP correlates with cortical atrophy, particularly in the entorhinal cortex and other AD-vulnerable regions [143], reinforcing its value as a progression marker.

Beyond AD, hFABP has been investigated as a potential biomarker to distinguish between AD, DLB, and PD [141,144]. Studies indicate that CSF hFABP levels are significantly higher in AD and DLB than in PD, suggesting its use in the differential diagnosis. However, unlike CSF hFABP levels, blood hFABP levels were not correlated with AD. Additionally, an earlier study proposed that the CSF hFABP/tau ratio may help differentiate AD from DLB [145].

Recent studies have linked FABP to neuroinflammation. CSF analysis revealed correlations between hFABP and inflammatory markers, including sTREM2, MIF, VEGF-R, and sVCAM-1, suggesting that neuronal damage in AD may be accompanied by chronic inflammation [146]. This evidence supports the hypothesis that hFABP is involved in neurodegenerative processes beyond lipid metabolism, including inflammation-mediated neuronal damage.

Overall, hFABP has emerged as a promising biomarker for AD, with studies supporting its role in early diagnosis, progression monitoring, and differentiation from other neurodegenerative disorders. However, further large-scale clinical validation is required to establish the diagnostic and prognostic utility of hFABP levels in AD.

### 5.9. Monocyte Chemoattractant Protein-1

MCP-1, also known as CCL2, is a chemokine involved in immune cell recruitment and inflammatory regulation [147] (Table 10). It plays a critical role in guiding monocytes and lymphocytes to sites of inflammation, affecting both immune responses and BBB permeability [148].

Several studies have implicated MCP-1 in AD development. One of the first investigations focused on the -2518 A/G polymorphism of MCP-1, identifying the GG variant as a significant risk factor for AD in an Italian cohort [149]. This association appears to be independent of APOE ε4 and other pro-inflammatory genetic variants, reinforcing MCP-1’s potential role in AD pathogenesis. Subsequent studies in transgenic AD mouse models have demonstrated that MCP-1 overexpression leads to excessive microglial accumulation and increased Aβ deposition, exacerbating disease progression [150].

The relevance of MCP-1 as a biomarker was further explored through CSF analysis in individuals with MCI [151]. The findings indicate that elevated MCP-1 levels in the CSF correlate with faster cognitive decline, suggesting that this chemokine may accelerate neurodegeneration in individuals with altered Aβ metabolism. Similarly, post-mortem analyses of AD brain tissues revealed significantly elevated MCP-1 levels compared with the controls, reinforcing its role as a potential disease marker [148].

A longitudinal study on patients with AD and MCI found that high plasma MCP-1 levels correlated with faster cognitive decline over two years, reinforcing its potential role as a prognostic biomarker [152]. Additionally, the CCR2 rs1799864 polymorphism has been associated with higher MCP-1 levels in AD patients, suggesting a genetic interaction in MCP-1 regulation.

More recently, a study demonstrated that elevated MCP-1 levels in the blood increase the risk of AD in individuals carrying HLA-DRB1 rs9271192-C and APOE ε4 risk alleles. A synergistic effect between MCP-1 and C-reactive protein (CRP) was observed, further compromising BBB integrity and exacerbating neuroinflammation [153]. The disruption of BBB permeability facilitates the entry of peripheral immune cells into the brain, leading to worsened neuronal damage.

The overexpression of MCP-1 has also been linked to aggravated tau pathology, characterized by increased neuroinflammation and hyperphosphorylated tau deposition in AD mouse models [154]. This process is accompanied by T cell infiltration in the meninges, indicating that MCP-1 may drive tauopathy through a neuroinflammatory mechanism.

Collectively, these findings suggest that MCP-1 is a promising biomarker for AD, as elevated levels in the blood and CSF are associated with greater disease risk, accelerated cognitive decline, and more severe neurodegeneration. However, further research is required to fully elucidate MCP-1’s mechanistic involvement in AD pathophysiology.

### 5.10. Transactive Response DNA Binding Protein 43 kDa 

TDP-43 is a nuclear protein encoded by the TARDBP gene on chromosome 1 (1p36.22) [155] (Table 11). It plays a crucial role in various cellular functions, including mRNA splicing regulation, RNA transport, and microRNA biogenesis. TDP-43 regulates over 4000 mRNA transcripts and maintains self-regulation through a negative feedback mechanism that destabilizes its mRNA. Additionally, it contributes to stress response pathways by forming stress granules that modulate protein synthesis under cellular stress conditions, such as heat shock, oxidative stress, and viral infections.

TDP-43 has been identified as a key pathological protein in several NDs, including amyotrophic lateral sclerosis (ALS) and frontotemporal lobar degeneration (FTLD). Recently, its involvement in AD has been studied extensively [156,157,158].

Post-mortem analyses have identified TDP-43 aggregates in approximately 57% of AD brains, suggesting a strong association between TDP-43 pathology and disease progression [156,159,160]. The abnormal accumulation of TDP-43 follows a characteristic spreading pattern in the brain, beginning in the amygdala and progressively affecting the entorhinal cortex, hippocampus, and neocortical regions [161]. This distribution mirrors the propagation of tau pathology in AD patients.

The presence of TDP-43 pathology in AD is correlated with accelerated cognitive decline, increased brain atrophy, and greater functional impairment compared to patients with only amyloid and tau pathology [156,162]. Furthermore, TDP-43 accumulation is linked to advanced neurodegeneration and amyloid deposition, highlighting its critical role in AD progression [163].

At the molecular level, the interaction between TDP-43 and key proteins involved in AD suggests that it may play an active role in disease progression [164,165]. Experimental studies have indicated that Aβ42 can trigger pathological modifications of TDP-43, including its phosphorylation and cytosolic accumulation, independently of tau pathology. In transgenic AD mouse models, increased Aβ expression correlates with enhanced TDP-43 pathology and Aβ clearance prevents these changes. These findings suggest that Aβ plays a central role in modulating TDP-43 pathology, highlighting the potential overlap between the AD and ALS-FTLD mechanisms. In contrast, TDP-43 depletion appears to enhance microglial responses, aid in amyloid clearance, and contribute to synaptic loss and neurodegeneration [166].

However, the relationship between TDP-43 and tau remains complex. TDP-43 interacts with tau and accumulates in the cytoplasm in the presence of tau oligomers. In AD, ALS, and FTD, TDP-43 may exacerbate tau pathology through cross-seeding mechanisms, promoting tau aggregation and neurotoxicity [167,168].

Another crucial aspect is the association between TDP-43 and the APOE4 genotype [155,169]. Studies indicate that patients with APOE4 and TDP-43 pathology exhibit faster cognitive decline than those with amyloid pathology alone, suggesting that TDP-43 may represent an additional risk factor for AD.

Despite increasing evidence of TDP-43’s role in AD, its validation as a diagnostic biomarker remains ongoing. Currently, TDP-43 pathology can only be diagnosed postmortem, which limits its clinical application. However, identifying fluid biomarkers of TDP-43 could significantly enhance the A,T(N) classification system, providing an independent indicator of dementia risk and enabling a more accurate diagnosis of AD and mixed neurodegenerative conditions [170,171]. Having a TDP-43 biomarker could also help differentiate pure AD cases from those with comorbid conditions, such as Limbic-predominant Age-related TDP-43 Encephalopathy (LATE). LATE primarily affects older adults and shares neuropathological features with AD but presents a distinct clinical progression [162].

The growing understanding of TDP-43 pathology and its interaction with key AD-related processes suggests that it could serve as a complementary biomarker for the diagnosis and prognosis of AD. Further research is needed to establish reliable detection methods and clarify their exact contribution to neurodegeneration.

### 5.11. Brain-Derived Neurotrophic Factor 

BDNF is a critical neurotrophin involved in the development, maintenance, and plasticity of neural networks [172] (Table 12). As a member of the neurotrophin family, it plays a key role in neuroprotection, neuronal differentiation, and survival. The BDNF gene, located on chromosome 11, comprises 11 functional exons and 9 promoters, each responsible for expressing specific BDNF isoforms that regulate various brain and behavioral functions [173]. The two most studied forms of BDNF are pro-BDNF, an inactive precursor, and mature BDNF (mBDNF), the active form responsible for most neurotrophic functions. BDNF exerts its effects by binding to the TrkB receptor and activating intracellular signaling pathways, such as PI3K/Akt, MAPK/ERK, and PLCγ, which are involved in cell growth, survival, and synaptic plasticity [174].

The role of BDNF as a biomarker for AD has gained significant attention because of its association with neurodegeneration. Studies indicate that BDNF levels in the brain, blood, and CSF are significantly lower in AD patients, and that higher BDNF levels correlate with better cognitive function [175,176]. BDNF is particularly abundant in the hippocampus, prefrontal cortex, and entorhinal cortex, regions that show early degeneration in AD [177].

One of the most studied genetic variations in BDNF is the Val66Met polymorphism, which involves the substitution of valine with methionine in the prodomain of the protein. This polymorphism has been linked to reduced BDNF secretion and increased risk of cognitive decline, particularly in women [178,179].

BDNF is involved in multiple molecular pathways that are implicated in AD. This reduction was associated with Aβ accumulation, tau phosphorylation, and neuroinflammation [180]. Activation of the TrkB receptor by BDNF has been shown to reduce tau phosphorylation via the PI3K/Akt pathway, while BDNF depletion, linked to Aβ accumulation, results in synaptic dysfunction and neurodegeneration [181,182]. Additionally, BDNF upregulation via the ERK/CREB pathway prevents dendritic atrophy and neuronal loss caused by Aβ toxicity [183].

BDNF can be measured in the CSF, serum, and plasma as a potential biomarker for AD. Studies have reported lower CSF BDNF levels in patients with AD, but the results vary owing to methodological challenges, particularly in distinguishing pro-BDNF from mBDNF [184]. In serum and plasma, higher BDNF levels correlate with better cognitive function and a lower risk of AD, suggesting its potential role as a diagnostic and prognostic biomarker [185,186].

From a therapeutic perspective, enhancing BDNF levels has been proposed as a strategy to slow AD progression. BDNF promotes the non-amyloidogenic pathway of APP, activating α-secretase and reducing Aβ production [187]. Additionally, BDNF regulates SORL1, which influences APP trafficking and metabolism, thereby limiting Aβ accumulation [172]. BDNF attenuates AD-related neuroinflammation by inhibiting the TLR4/NF-κB pathway, which is activated by lipopolysaccharide (LPS) [188,189].

Despite their potential as biomarkers and therapeutic targets, several challenges remain to be overcome. The variability in BDNF levels across different biological fluids, along with difficulties in distinguishing its isoforms, necessitates the development of standardized measurement methods [184]. Furthermore, BDNF’s limited ability to cross the BBB limits its therapeutic application. Innovative strategies such as nanobiodegradable carriers and intranasal administration are being explored to enhance BDNF delivery to the brain [172].

BDNF is a promising biomarker for diagnosing and monitoring AD, as well as a potential therapeutic target. However, further research is needed to clarify its role in neurodegeneration, optimize measurement techniques, and develop effective delivery methods for clinical applications in AD management.

## 6. Conclusions

AD is a complex and progressive neurodegenerative disorder that is a major public health concern. In recent years, substantial advancements have been made in elucidating the molecular mechanisms underlying AD, enabling the development of novel diagnostic frameworks, such as the ATX(N) classification system. These biomarkers not only enhance diagnostic accuracy but also provide critical insights into the pathophysiological progression of the disease.

The transition from a symptom-based diagnosis to a biomarker-driven approach represents a significant paradigm shift in AD research and clinical practice. Core biomarkers are now well-established as essential tools for tracking disease progression and staging. However, the addition of emerging biomarkers, such as SNAP-25, GFAP, YKL-40, TREM2, and IGF-1, has enhanced our knowledge of the disease, highlighting new aspects such as synaptic dysfunction, neuroinflammation, and BBB disruption. Many of these new biomarkers can now be measured in blood samples or neuron-derived exosomes, making diagnosis less invasive and more accessible and allowing for earlier detection and possibly large-scale screening.

Among these emerging biomarkers, SNAP-25 appears particularly promising. This synaptic protein increases in the CSF during the early stages of the disease, reflecting synaptic damage before symptoms appear. It can also be detected in blood-derived exosomes, which makes it a valuable and easy-to-access biomarker. Similarly, GAP-43, which is linked to amyloid and tau pathologies, may help to monitor disease progression. GFAP, a marker of astrocyte activation, has shown high sensitivity and specificity, particularly in plasma tests, and its inclusion in the updated 2024 diagnostic criteria highlights its increasing clinical relevance.

Other biomarkers, such as TREM2, PGRN, and MCP-1, offer important insights into the role of the immune and inflammatory responses in AD. However, their diagnostic use remains limited owing to their low specificity and high variability between individuals.

Overall, the identification of new biomarkers is important for improving the diagnosis and management of AD. Although many emerging biomarkers still need more research, their strong association with the key pathological mechanisms of AD makes them promising candidates for inclusion in the current diagnostic and management criteria. In conclusion, this approach makes it possible to shift from a diagnosis solely based on clinical symptoms to a more specific biology-based diagnosis.

Despite the great potential of these emerging biomarkers, their use in everyday clinical practice is still limited by several practical issues. These include the lack of assay standardization, variability in pre-analytical handling, limited access to advanced technologies, and the absence of universally accepted reference ranges. Solving these problems is key to improving test reliability, standardization, and clinical accessibility in all settings.

In parallel, future research should focus on exploring key knowledge gaps. These include the need for large-scale, longitudinal studies to assess the diagnostic and prognostic accuracy of markers across disease stages, and the identification of optimal biomarker combinations for personalized risk stratification. Moreover, defining the evolution of these biomarkers over time and their utility in preclinical or atypical presentations will be critical. Incorporating such markers into updated diagnostic algorithms may ultimately lead to earlier detection, more precise classification, and improved patient outcomes.

## Figures and Tables

**Table 2:** Synaptosomal-associated protein 25 (SNAP-25).

	SNAP-25
**Sample type**	CSF and neuron-derived exosomes
**Diagnostic accuracy**	AUC values in various cohorts range from 0.80 to 0.90, indicating high diagnostic accuracy.
**Prognostic value**	It is a prognostic biomarker reflecting early synaptic dysfunction. Longitudinal studies have shown that higher SNAP-25 levels predict faster cognitive decline and conversion from MCI to AD.
**Advantages**	Early detection of synaptic dysfunction; high diagnostic accuracy; predictive of disease progression; correlates with established biomarkers; reflects core pathophysiology; potential for non-invasive testing; possible role in treatment monitoring.
**Limitations**	Lack of assay standardization; limited clinical availability; invasiveness of CSF sampling; lack of established cut-off values; nonspecific to AD; inter-study variability.

**Table 3:** Chitinase-3-like protein 1 (CHI3L1 or YKL-40).

	YKL-40
**Sample type**	CSF and blood
**Diagnostic accuracy**	Moderate diagnostic accuracy (AUC ~0.70–0.80). It enhances sensitivity to early disease stages and complements core CSF biomarkers.
**Prognostic value**	Correlates with disease progression and cognitive decline. CHI3L1 levels increase progressively from cognitively normal aging to MCI and AD dementia. This elevation appears to precede or accompany tau accumulation, suggesting that CHI3L1 reflects inflammatory responses downstream of amyloid deposition. Individuals with higher YKL-40 levels show greater rates of brain atrophy, especially in regions affected early by AD, such as the hippocampus.
**Advantages**	Elevated early in the disease course; predictive of disease progression; measurable in CSF and blood, supporting development of less invasive diagnostic approaches.
**Limitations**	Nonspecific to AD; inter-study variability; limited standardization across laboratories.

**Table 4:** Neuromodulin or GAP-43.

	GAP-43
**Sample type**	CSF and blood exosomes
**Diagnostic accuracy**	High diagnostic accuracy in CSF, with AUCs frequently exceeding 0.85. Its levels increase early in the disease continum, including preclinical and prodromal AD.
**Prognostic value**	Early indicator of disease progression and it is associated with cognitive decline, correlating with tau pathology, brain atrophy (especially hippocampal), and declines in memory and executive function over time. Individuals with elevated GAP-43 show faster cognitive deterioration in longitudinal studies.
**Advantages**	Reflects early synaptic dysfunction; high diagnostic accuracy; predictive of disease progression
**Limitations**	Limited availability in clinical practice; lack of standardized assays and cut-offs; limited blood-based data.

**Table 5:** Visinin-like protein 1 (VILIP-1 or VLP-1).

	VILIP-1
**Sample type**	CSF and Blood
**Diagnostic accuracy**	Good diagnostic accuracy for Alzheimer’s disease, with AUC values up to 0.89 in CSF.
**Prognostic value**	Elevated levels predict cognitive decline, disease progression, and brain atrophy, even in preclinical or early symptomatic stages. Its role in reflecting neuronal injury complements amyloid and tau pathology, supporting its integration into biomarker panels for longitudinal monitoring.
**Advantages**	Good diagnostic and prognostic performance; reflects neuronal injury.
**Limitations**	Variability across studies; reduced specificity (↑ in DLB and CJB).

**Table 6:** Progranulin (PGRN).

	PGRN
**Sample type**	CSF and cerebral tissue
**Diagnostic accuracy**	Limited diagnostic accuracy with AUC values being low to moderate (typically < 0.75), indicating limited standalone diagnostic accuracy.
**Prognostic value**	Modest prognostic value in Alzheimer’s disease, potentially reflecting neuroinflammatory burden and disease progression.
**Advantages**	Reflects microglial activation and lysosomal dysfunction; detectable in multiple biological fluids, which supports its future potential for less invasive testing.
**Limitations**	Low specificity to AD; variable results; limited diagnostic and prognostic accuracy alone; high inter-individual variability; lack of standardized assays and cut-offs.

**Table 7:** Triggering receptor expressed on myeloid cells 2 (TREM2).

	TREM2
**Sample type**	CSF and Blood
**Diagnostic accuracy**	Moderate diagnostic accuracy (AUC ~0.65–0.75), particularly in early symptomatic stages. While not suitable as a standalone diagnostic biomarker, it adds value to multiplex biomarker panels by capturing the microglial response associated with AD progression.
**Prognostic value**	It reflects microglial activation dynamics and may help predict the rate of disease progression, especially in the early symptomatic phase. Its prognostic utility is strongest when combined with other biomarkers in a multimodal framework.
**Advantages**	Reflects microglial activation; dynamic in early disease stages; potential prognostic utility.
**Limitations**	Nonspecific to AD; limited diagnostic accuracy alone; assay standardization needed; limited data in blood.

**Table 8:** Insulin-like growth factor-1 (IGF-1).

	IGF-1
**Sample type**	CSF and blood
**Diagnostic accuracy**	Limited diagnostic accuracy for AD, with inconsistent findings and AUC values generally <0.70. While biologically relevant to AD pathogenesis, it is not suitable as a standalone diagnostic marker, but may have utility in multi-biomarker panels exploring metabolic dysfunction.
**Prognostic value**	Lower IGF-1 levels in plasma or CSF have been associated with faster cognitive decline and greater risk of progression from MCI to AD in some longitudinal studies. IGF-1 may modulate tau phosphorylation and Aβ clearance, indirectly influencing neurodegeneration. Reduced IGF-1 is linked to hippocampal atrophy and decreased glucose metabolism on PET imaging, both biomarkers of AD severity and progression. It may help identify patients with metabolic-driven AD phenotypes.
**Advantages**	Reflects metabolic dysfunction; involved in multiple AD-related pathways; non-invasive measurement.
**Limitations**	Low disease specificity; nonspecific to AD; lack of standardized cut-offs.

**Table 9:** Heart-type fatty acid-binding protein (FABP).

	FABP
**Sample type**	CSF
**Diagnostic accuracy**	Good diagnostic accuracy with AUC values ranging from ~0.80 to 0.90. It may help differentiate AD from other neurodegenerative conditions, although its specificity remains under investigation.
**Prognostic value**	Higher CSF hFABP levels are associated with faster cognitive decline, greater brain atrophy, and increased risk of conversion from MCI to AD. Correlates with other markers of neurodegeneration (e.g., tau, YKL-40) and inflammatory changes, supporting its use as a marker of disease progression. May be useful in identifying individuals with rapidly progressing AD phenotypes.
**Advantages**	Good diagnostic and prognostic accuracy.
**Limitations**	Limited clinical availability; measured in CSF; lack of disease specificity; no established clinical cut-off values.

**Table 10:** Monocyte chemoattractant protein-1 (MCP-1).

	MCP-1
**Sample type**	CSF and blood
**Diagnostic accuracy**	Modest diagnostic accuracy (AUC ~0.65–0.75).
**Prognostic value**	Associated with MCI-to-AD progression and greater brain pathology. Elevated MCP-1 levels are linked to APOE ε4 carriers, suggesting it may help identify individuals with genetic susceptibility to inflammatory AD phenotypes.
**Advantages**	Detectable in blood and CSF; reflects neuroinflammation and immune activation; potential role in genetic risk stratification.
**Limitations**	Not disease-specific; modest diagnostic performance; lack of standardized cut-offs

**Table 11:** Transactive response DNA binding protein 43 kDa (TDP-43).

	TDP-43
**Sample type**	Currently, there is no validated fluid biomarker (e.g., in CSF or blood) for TDP-43 in clinical use.
**Diagnostic accuracy**	Diagnosis of TDP-43 pathology is limited to post-mortem brain tissue via immunohistochemistry. Thus, its diagnostic accuracy in living patients is currently unknown, and research is ongoing to develop fluid-based assays (e.g., plasma exosomal or phosphorylated TDP-43).
**Prognostic value**	Despite the absence of a reliable fluid test, neuropathological studies show that TDP-43 aggregates are strongly associated with faster cognitive decline, greater hippocampal atrophy, and worse clinical outcomes in AD patients. TDP-43 pathology often coexists with tau and amyloid pathology, but adds independent prognostic value, particularly in limbic-predominant age-related TDP-43 encephalopathy, which mimics AD clinically.
**Advantages**	Strong prognostic value; found in up to 50% of AD cases, especially in older individuals and APOE ε4 carriers, making it a highly relevant biomarker for late-stage and mixed pathology cases; supports recognition of limbic-predominant age-related TDP-43 encephalopathy.
**Limitations**	No validated in vivo biomarkers; overlaps with other neurodegenerative diseases.

**Table 12:** Brain-derived neurotrophic factor (BDNF).

	BDNF
**Sample type**	CSF and blood
**Diagnostic accuracy**	Modest accuracy, with AUC values typically ranging from 0.65 to 0.75, indicating limited standalone diagnostic value.
**Prognostic value**	Lower BDNF levels are associated with faster cognitive decline, particularly in episodic memory and executive function. Reduced BDNF may predict conversion from MCI to AD, especially when combined with other biomarkers (e.g., Aβ42, tau). BDNF Val66Met polymorphism, which impairs BDNF signaling, has been linked to greater hippocampal atrophy and poorer cognitive outcomes in individuals at risk for, or diagnosed with, AD.
**Advantages**	Early indicator of neurodegeneration; prognostic utility; non-invasive measurement.
**Limitations**	Low diagnostic accuracy; high variability; lack of standardized cut-offs.
